# Rapid and persistent selection of the K103N mutation as a majority quasispecies in a HIV1-patient exposed to efavirenz for three weeks: a case report and review of the literature

**DOI:** 10.4076/1752-1947-3-9132

**Published:** 2009-09-18

**Authors:** Ennio Polilli, Giustino Parruti, Luana Cosentino, Federica Sozio, Annalisa Saracino, Augusta Consorte, Gioacchino Angarano, Francesco Di Masi, Elena Mazzotta, Paolo Fazii

**Affiliations:** 1 Clinical Pathology Unit, Ospedale Civile "Spirito Santo", Via Fonte Romana 8, 65124 Pescara, Italy; 2 Infectious Disease Unit, Ospedale Civile "Spirito Santo", Via Fonte Romana 8, 65124 Pescara, Italy; 3 Infectious Disease Department, University of Foggia, "Ospedali Riuniti", Via L. Pinto 1, 71100 Foggia, Italy

## Abstract

**Introduction:**

Selection of the K103N mutation is associated with moderately reduced in vitro fitness of HIV. Strains bearing K103N in vivo tend to persist, even in the absence of additional drug pressure, as minority quasispecies, often undetectable in genotyping resistance testing assays, performed at standard conditions. Here, we report on the rapid and long lasting selection of a K103N bearing strain as the dominant quasispecies after very short exposure to efavirenz in vivo.

**Case presentation:**

A 55-year-old Caucasian man was switched to efavirenz, zidovudine and lamivudine in February 2003, while on viral suppression in his first-line highly active anti-retroviral treatment regimen. One month later, he reported inconsistent adherence and his viremia level was 5700 c/mL. He did not attend further checkups until September 2005, when his viral load was 181,000 c/mL. The patient reported interrupting his medications approximately three weeks after simplification. The genotyping resistance testing assay was performed both on HIV RNA and HIV DNA from plasma, yielding an identical pattern with the isolate presence of the K103N mutation in the prevalent strain.

**Conclusion:**

Persistence of the K103N mutation as a majority quasispecies may ensue after a very short exposure to efavirenz. Our case would therefore suggest that the presence of the K103N mutation should always be ruled out by genotyping resistance testing assays, even after minimal exposures to efavirenz.

## Introduction

Non-nucleoside reverse transcriptase inhibitor (NNRTI)-based highly active anti-retroviral treatment (HAART) regimens are characterized by a good tolerance of relatively low levels of adherence to therapy, although resistance selection may ensue when adherence drops below the 75% threshold [1,2]. First generation NNRTIs have a low genetic barrier and the K103N mutation is able to confer class resistance after selection [3,4]. As efavirenz (EFV) and nevirapine (NVP) have a much longer plasma half-life than their companion nucleoside reverse transcriptase inhibitors (NRTIs), residual NNRTI monotherapies associated with structured or non-structured therapy interruptions may favor K103N selection. Although selection of the K103N mutation is associated with moderately but significantly reduced in vitro fitness [5], strains bearing K103N in vivo tend to persist even in the absence of additional drug pressure. K103N persistence, however, is more frequently observed as a minority quasispecies, often undetectable in GRT (Genotypic antiretroviral Resistance Testing) assays performed at standard conditions.

A single dose of NVP administered during delivery in pregnant women to prevent mother from transmitting HIV to their babies was able to select K103N mutant strains, which could persist as minority quasispecies for as long as five years after drug exposure [6]. Persistence of NNRTI-induced mutations, and particularly persistence of the K103N mutation, has also been observed in patients on treatment interruptions after failure of NNRTI-based regimens, as well as in patients on failing protease inhibitor (PI)-based regimens after previous NNRTI failures [7]. In this report, we describe the case of a 55-year-old HIV-infected man, for whom a quickly selected and long-term persisting K103N mutation was detected after a time of exposure to EFV as short as three weeks. The K103N bearing strain persisted as the dominant quasispecies for over three years in the absence of any further drug pressure, as documented by a GRT assay performed at the end of a long-lasting treatment interruption.

## Case presentation

A 55-year-old Caucasian male patient infected with HIV for more than 10 years after heterosexual exposure, CDC class C since 1999 due to pneumocystis jiroveci pneumonia, was put on his first-line HAART regimen in November 1998, with indinavir, zidovudine (AZT) and lamivudine (LMV). His virological response was prompt and long lasting. Pre-HAART viremia level was 215,000 c/mL (5,3Log_10_, no GRT assay was performed at that time), pre-HAART CD4+ T-lymphocytes were 17/µL. After viral suppression, his CD4+ T-cell counts very slowly improved, approaching the 200/µL threshold only after 28 months of treatment. Therefore, his HAART scheme was not simplified, with no signs of drug-related toxicity ensuing. In February 2003, however, after approximately four years of treatment, the patient declared his intention to change his treatment drugs. According to the patient, he experienced treatment-related fatigue and had recent adherence failures. His viral load at that time, however, was still undetectable. His treatment was therefore switched to EFV, AZT and LMV.

**Figure 1:**
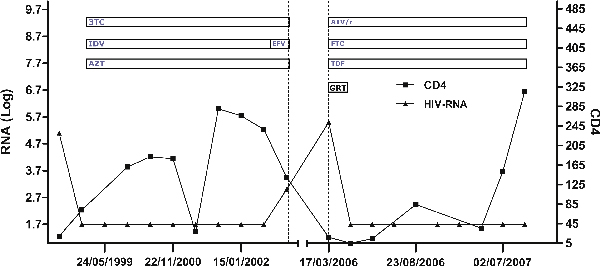
**Human immunodeficiency virus viremia, CD4+ T-cell counts and therapy during follow-up (3TC Lamivudine, AZT Zidovudine, ATV Atazanavir, IDV Indinavir, EFV Efavirenz, TDF Tenofovir, FTC Emtricitabine, GRT Genotyping Resistance Testing)**.

One month later, during his next scheduled control, his viremia was 5700 c/mL (3,7 Log_10_); he reported inconsistent adherence. After that, he did not show up until March 2006. He was hospitalized due to clinical progression (high fever and remarkable weight loss), with a discharge diagnosis of disseminated atypical mycobacteriosis. On admission, CD4+ T-cell counts were 12/µL, and his viral load was 181.000 c/mL (5,2 Log_10_). The patient reported interruption of all his medications a few days after his last check in 2003, approximately three weeks after therapy simplification (Figure 1). Neither a pre-NNRTI GRT assay was available at that time, nor samples of plasma drawn in advance of the NNRTI-based treatment, had been stored. GRT assays, however, were performed both on HIV RNA and on HIV DNA from peripheral blood. These proved that HIV infection was due to clade B HIV-1, both yielding an identical sequence pattern, with the isolate presence of the K103N mutation in the prevalent strain (Table 1). The patient was put on a new PI-based HAART regimen including boosted atazanavir, tenofovir and emtricitabine, reaching a prompt and complete (<40 c/mL) viral suppression after two weeks.

**Table 1:** GRT of proviral and plasma HIV, as interpreted by the Stanford HIV database algorithm (as of March, 2006)

	HIV-DNA resistance mutations	HIV-RNA resistance mutations
PI Major Resistance Mutations:	None	None
PI Minor Resistance Mutations:	L10V	None
PI Other Mutations:	I15V	V11FV
	R41K	E35D
	L63P	N37NS
		R41K
		P44LP
		R57K
		L63P
		G94GRS
NRTI Resistance Mutations:	None	None
NNRTI Resistance Mutations:	K103N	K103N
RT Other Mutations:	K64R R211K V245Q	K30Q, K32GRS, A33C, L34T, V35L, E36N, I37F, K46KT, V148EV, Q174LQ, R211K, V245Q

## Discussion

Assuming that HIV replication relapsed seven to 14 days after interruption of the EFV-containing HAART regimen, our patient was likely exposed to a residual monotherapy with EFV for some one to two weeks, as pharmacokinetics data on EFV indicate a serum half-life of 14.6-167.6 hours, significantly longer than that of AZT (1 to 2 hours) and LMV (8 hours). Furthermore, chromatographic assays for EFV, performed seven days after the last dose, demonstrated sub-therapeutic concentrations of EFV in plasma (70-720 ng/mL) [8]. A GRT had not been performed at the time of first HIV diagnosis, so straightforward evidence could not be provided to exclude the presence of the K103N mutation in our patient in advance of his first line HAART.

He first presented in our ward in 2000, with very advanced HIV infection (Nadir CD4 counts = 15/mm^3^) and pneumocystis jiroveci pneumonia. He never travelled abroad, and reported risky heterosexual behaviors up to some 10 years before presentation. As a consequence, although both the existence [9] and the transmission [10] of HIV strains carrying the K103N mutation in NNRTI-naïve patients have been extensively documented, it is unlikely that our patient may have been infected with an HIV strain carrying the K103N mutation, as the widespread use of NNRTIs in Italy began after 1996. Therefore, it is more likely that exposure to EFV monotherapy at the time of therapy interruption selected the K103N mutation, which persisted as the prevalent quasispecies for over 3 years of continual viral replication, in the absence of selective pressure.

Our experience is therefore very similar to the observed scenario of HIV-infected women exposed to a single dose of NVP for prevention of mother-to-child HIV transmission. Reports on such women, however, indicate that the K103N quasispecies were present as a minority quasispecies in most described cases [6,11,12]. Similarly, by means of high sensitivity sequencing techniques, Palmer et al. reported on the persistence of K103N mutation after EFV interruption in patients failing to EFV-containing regimens, documenting variable decay kinetics for the K103N containing quasispecies. Such quasispecies most frequently decayed and disappeared after a few weeks, but less frequently so, after a few months. In one patient, strains carrying K103N mutation persisted for six years as dominant quasispecies, in the absence of selective pressure, after administration of EFV for a year and a half. In such a case, the authors postulated a possible eradication of the wild type quasispecies before HAART failure [13]. Capetti et al. reported the case of a HIV-infected woman who, seven years after exposure to a failing loviride-containing HAART regimen, presented the K103N mutation at a later viral relapse, without further exposure to any NNRTI selective pressure for as long as seven years after loviride. This patient, however, had been kept under prolonged viral suppression on protease inhibitor (PI)-containing HAART regimens, likely limiting viral replicative competition [14]. In our patient, persistence of the K103N-containing quasispecies ensued after a short exposure to EFV, in the absence of any further selective pressure. Therefore, it likely depended on a relatively small decrease in viral fitness of this prevalent quasispecies relative to wild type.

It is now well-known that NNRTIs bind a hydrophobic site adjacent with the enzyme active site of HIV reverse transcriptase, inducing an allosteric modification of the active site influencing enzyme performance [15]. Collins et al., applied a fitness assay to several NNRTI drug-resistant quasispecies, demonstrating that the wild type had a better fitness than K103N-bearing quasispecies, and that the fitness rank for other NNRTI mutation-carrying quasispecies was 181C > 190A > 188C > 106A.

Furthermore, they demonstrated that double and triple NNRTI mutation-carrying quasispecies had worse fitness than single mutation carrying quasispecies [5]. Along a different line of evidence, Hatano et al. recently demonstrated that failing NNRTI-based HAART regimens do not confer clinical residual advantage, at variance with PI-based failing HAART regimens. This observation would suggest that unlike PI-induced mutations, NNRTI-induced mutations do not significantly alter fitness of mutation-carrying quasispecies [16].

This scenario confirms the result of Fox et al. 2008, and highlights the need to interrupt NNRTI-based regimens wisely. Patients of the SMART study interrupting NNRTI-based regimens restarted later on the same regimen. Fox et al. demonstrated that patients on staggered interruptions (interruption of NRTIs one week after that of the NNRTI) or switched interruptions (replacement of the NNRTI with a boosted PI for a short period) of all drugs were less likely than those using simultaneous interruptions to have any NNRTI mutation in their GRT performed two months following the treatment interruption. They also demonstrated that patients who carried a NNRTI-mutation after the interruption achieved viral resuppression significantly less frequently than those who did not have any mutation in their resistance test [17].

## Conclusion

Our experience, although similar to that reported by Palmer et al. on one of their patients, would indicate that persistence of the K103N mutation as a majority quasispecies may ensue after a very short exposure to EFV, in the absence of wild type depletion, likely providing relative replicative advantage.

Our case therefore suggests that the presence of the K103N mutation should be ruled out even after minimal exposures to EFV, reinforcing at the same time the need for a systematic evaluation of GRT in naïve patients and after any type of interruption of NNRTI-based HAART regimens, as well as the need for a wise strategy of NNRTI interruption.

## Abbreviations

μL: microliter; EFV: efavirenz; GRT: genotypic antiretroviral resistance testing; HAART: highly active antiretroviral therapy; mL: milliliter; ng: nanogram; NNRTI: non-nucleoside reverse transcriptase inhibitor; NVP: nevirapine; PI: protease inhibitor.

## Competing interests

The authors declare that they have no competing interests.

## Consent

Written informed consent was obtained from the patient for publication of this case report and any accompanying images. A copy of the written consent is available for review by the Editor-in-Chief of this journal.

## Authors' contributions

EP, GP, AC, FDM and PF ideated this case report after observation of the GRT results, and did most of the writing, supported by FS and EM; LC and EP performed RNA sequencing and sequence interpretation; AS and GA contributed the DNA results and critical counselling.
